# Ball-milled MoS_2_ with graphene shows enhanced catalytic activity for hydrogen evolution reaction

**DOI:** 10.1080/14686996.2024.2359360

**Published:** 2024-05-29

**Authors:** Linghui Li, Satish Laxman Shinde, Takeshi Fujita, Takahiro Kondo

**Affiliations:** a Graduate School of Pure and Applied Sciences, University of Tsukuba, Tsukuba, Japan; b Department of Materials Science, Institute of Pure and Applied Sciences, University of Tsukuba, Tsukuba, Japan; c Department of Physics, Indian Institute of Technology Hyderabad, Kandi, India; d School of Engineering Science, Kochi University of Technology, Kochi, Japan; e Tsukuba Research Center for Energy Materials Science, Institute of Pure and Applied Sciences and R&D Center for Zero CO_2_ Emission with Functional Materials, University of Tsukuba, Tsukuba, Japan; f Advanced Institute for Materials Research, Tohoku University, Sendai, Japan

**Keywords:** Molybdenum disulfide, hydrogen evolution reaction, electrocatalyst, edge site, 1T phase, sulfur vacancy

## Abstract

The hydrogen evolution reaction (HER) is an important phenomenon in water splitting. Consequently, the development of an active, earth-abundant, and inexpensive HER catalyst is highly desired. MoS_2_ has drawn considerable interest as an HER catalyst because it is composed of non-precious metal and exhibits high catalytic activity in the nanosheet form. In this study, size-controlled MoS_2_ particles were synthesized by ball milling. The as-prepared samples exhibited significantly enhanced electrochemical and catalytic properties compared to those of pristine bulk MoS_2_. Furthermore, the HER activity improved further upon the introduction of graphene into the as-prepared ball-milled samples. In particular, the MoS_2_ sample ball-milled for 12 h mixed with graphene exhibited optimal performance, showing an overpotential (160 mV at 10 mA cm^−2^) that was ~ 335 mV lower than that of pristine bulk MoS_2_. The superior catalytic activity was ascribed to the exposed edge sites, sulfur vacancies, and 1T phase of MoS_2_, as well as the noteworthy fortifying effect of the electronically conductive flexible material, graphene. The results provide a promising strategy for its application as an efficient and stable HER catalyst.

## IMPACT STATEMENT

Li et al. reported MoS_2_-based electrocatalysts for the hydrogen evolution reaction. The superior catalytic activity was ascribed to exposed edge sites, sulfur vacancies, 1T phase, and the effect of graphene.

## Graphical abstract

**Figure:**
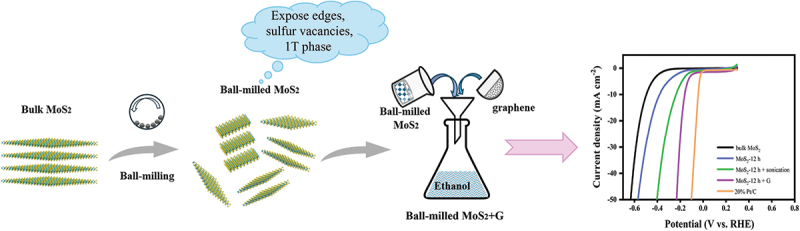

## 1. Introduction

The adverse impact of fossil fuels on the environment and the need to sustainably advance human society have led to a recent focus on replacing fossil fuels with renewable energy [1]. To that end, hydrogen has been promoted as an ideal energy carrier since the early 1970s because of its high weight energy density and zero CO_2_ emissions [2]. Hydrogen can store the energy generated by renewable sources in chemical bonds and convert it back into electricity when needed through fuel cells or other devices. Hydrogen is mainly present in compounds such as hydrocarbons and water on our planet and is primarily obtained through energy-intensive greenhouse-gas-emitting processes, such as the steam reforming of hydrocarbons [3]. However, achieving sustainable and economically feasible hydrogen production on an industrial scale is a major challenge [4]. One way to address this issue is to decompose water through electrolysis or photocatalysis, using renewable solar energy to separate water molecules directly or indirectly into their components [5,6].

The hydrogen evolution reaction (HER) is an extensively studied electrochemical process that involves the cathodic half-reaction of water decomposition [7]. It plays a crucial role in various energy-conversion devices, including hydroelectric and artificial photosynthetic cells. As its name suggests, the HER involves the reduction of protons or water molecules, leading to the release of gaseous hydrogen, as follows [8]:

(1.1) $$2H_{\left(aq\right)}^{+}+2e^{-}\to H_{2\left(g\right)},$$

where (*aq*) and (*g*) denote the form of H_3_O^+^ species in the aqueous solution and the gas phase, respectively. The standard reduction potential of the HER is defined as $E_{H^{+}/H_{2}}^{0}=0V$ *versus* a normal hydrogen electrode (NHE) at pH 0. However, similar to many chemical reactions, the electrochemical processes occurring during the HER must overcome a certain activation energy barrier (that is, the overpotential) to proceed. Electrocatalysts are typically required to reduce the overpotential, thereby improving the reaction rate and efficiency.

High-performance catalysts for the critical electrochemical HER should minimize the overpotential and thereby enhance its efficiency. Pt-group metals are considered remarkably efficient HER electrocatalysts. However, the development of HER catalysts with exceptional activity using materials that are more readily available and cost-effective is challenging [9].

Molybdenum disulfide (MoS_2_), which has been widely studied as a hydrodesulfurization catalyst, has recently garnered attention as an HER electrocatalyst because of its high catalytic activity [10–12]. Both computational and experimental findings have confirmed that the HER activity originates from the edges of the MoS_2_ plates [13,14], whereas their basal planes are catalytically inactive. Additionally, recent studies have reported other catalytic active sites of MoS_2_ toward the HER. For example, MoS_2_ with the 1T structural phase exhibits high catalytic activity, even with substantial oxidation at the edge sites [12,15,16]. Furthermore, the catalytic activity of a MoS_2_ film increases with decreasing thickness and is potentially superior to that of edge-rich pyramidal MoS_2_ nanosheets [17]. In addition to the edge sites, the sulfur vacancies of MoS_2_ provide another set of major catalytically active sites for the HER [15,18]. Consequently, the exposure of more edges, converted to the octahedral (1T) structural phase, and sulfur vacancy engineering are expected to be viable strategies for synthesizing MoS_2_ with higher catalytic performance than that of bulk MoS_2_ [13,16,19,20].

One limitation of MoS_2_ catalysts is that their active sites are limited to edges [21]. To fully harness the potential of MoS_2_-based catalysts, there is a pressing need to increase the number of active sites, enhance the activity of these catalytic sites, and improve the electrical connections between the active sites and catalyst substrate [22]. One approach to optimizing charge transfer involves the use of a graphene support, which has been demonstrated to significantly enhance the HER kinetics [12].

MoS_2_ belongs to the category of quasi-two-dimensional transition metal dichalcogenides with a layered structure, and has recently gained significant attention owing to its unique electronic, optical, optoelectronic, and catalytic properties [23]. In its bulk form, MoS_2_ is an indirect-bandgap semiconductor with an energy gap of 1.29 eV, and it comprises multiple layers of S – Mo – S bonds, held together by weak van der Waals interactions. Each layer comprises a central plane of Mo atoms sandwiched between two S atoms, with strong covalent bonds and dipole formation between the positively charged Mo atoms and negatively charged S atoms. The Mo atoms in MoS_2_ adopt two different coordination modes: 1T phase and trigonal prismatic (2 H and 3 R phases), with the 2 H phase being the most common in bulk MoS_2_. Notably, the crystal system can be exfoliated into individual layers owing to the weak van der Waals forces holding the S – Mo – S layers together [16,24]. These individual layers exhibit properties that differ significantly from those of the bulk material. However, although the conversion of 2 H MoS_2_ into the 1T phase can lead to enhanced catalytic performance in the HER [25,26], the precise mechanisms underlying this improvement are not fully understood.

Various methods have been developed to chemically exfoliate bulk MoS_2_ [16,24,25,27]. One approach involves the use of Li intercalation to construct single- or few-layered structures [23]. Alternatively, a bottom-up approach has been pursued to directly synthesize MoS_2_ nanostructures with a high density of edge sites [20,22]. Other methods include the chemical exfoliation of bulk MoS_2_ by sonication and solvent utilization [24,28]. Notably, all these methods involve wet-chemical processing schemes and may require potentially hazardous chemicals. Furthermore, chemical exfoliation tends to yield a minuscule amount of exfoliated material. Additionally, scaling up these methods can be challenging in terms of economics, environmental concerns (for example, Li intercalation and solvent use), and technological feasibility (for example, sonication) [19]. In this context, ball milling, a simple and efficient method, shows promise for improving the catalytic properties of bulk MoS_2_ [10,19].

In this study, a straightforward and scalable method based on dry ball milling was adopted to synthesize size-controlled MoS_2_ with significantly enhanced electrochemical catalytic properties. The original material – pristine bulk MoS_2_ powder – was not subjected to any chemical reactions or agents. Comprehensive characterization was performed using techniques such as scanning electron microscopy (SEM), energy-dispersive X-ray (EDX) spectroscopy, and X-ray photoelectron spectroscopy (XPS) to track the morphological and chemical changes that occurred during ball milling. Additionally, the possible introduction of metallic impurities could affect the electrochemical and catalytic properties of the material was probed. Overall, this study was aimed at synthesizing size-controlled MoS_2_ with enhanced HER-catalyzing activity by increasing the number of edge sites, introducing the 1T phase, and introducing sulfur vacancies via the simple ball milling method, and examining the effects of each process step on the activity.

## 2. Materials and methods

### 2.1. Materials

MoS_2_ bulk powder, graphene nanoplates (GNPs; average number of layers: 5–7), Nafion (5 wt% in lower aliphatic alcohols and water), H_2_SO_4_ (97%), and commercial Pt/C (20 wt% loading; matrix activated carbon support) were purchased from Sigma-Aldrich (Japan). The solvents were purchased from commercial sources.

### 2.2. Synthesis of ball-milled MoS_2_

A planetary ball mill (Fritsch PL-7, Flitch, Japan) whose container (capacity: 20 cm^3^) and balls (diameter: 1 cm) were made of ZrO_2_ was used for ball milling. Bulk MoS_2_ (100 mg) and six balls were placed in a container, and milling was performed at atmospheric pressure, a temperature of ~ 300 K, and a rotation speed of 400 rpm. The sample was set at ball-milling container in air. Each cycle involved 5 min of rotation, followed by a rest period of 30 s (to prevent temperature increases during long-term operation). Ball milling was performed for 15 min, 30 min, 45 min, 1 h, 2 h, 4 h, 8 h, and 12 h. The ball-milled samples are denoted herein based on the ball-milling duration; for example, the MoS_2_ sample ball-milled for 30 min is named ‘MoS_2_-30 min’.

### 2.3. Preparation of “MoS_2_-ball milled + sonicated” ink

Ball-milled MoS_2_ powder (5 mg) was suspended in a mixture of ethanol (1 mL) and Nafion (50 µL), and the resulting sample was subjected to 2 h of bath sonication (500 W/60 Hz, AS ONE Ltd., Japan), yielding a sample denoted as ‘MoS_2_-ball milled + sonicated’ herein.

### 2.4. Preparation of “MoS_2_-ball milled + G” ink

Ball-milled MoS_2_ powder (5 mg) and GNPs (10 mg) were suspended in ethanol (1 mL) and then underwent 1 h of bath sonication (500 W/60 Hz, AS ONE Ltd., Japan). A Nafion solution (50 µL) was added to the resulting mixture to prepare the ink for electrochemical measurements, denoted herein as ‘MoS_2_-ball milled + G’.

### 2.5. Characterization

Powder X-ray diffractometry (XRD) was performed using a Rigaku MiniFlex instrument (Tokyo, Japan) with a Cu Kα X-ray source (λ = 1.540598 Å). Diffraction patterns were recorded using a D/teX Ultra silicon strip detector (Rigaku) at a speed of 0.05° s^−1^ up to 2*θ* = 80°, where *θ* is the angle of incidence. SEM and electron-probe microanalysis were performed using a JXA-8530F instrument (JEOL Ltd., Japan) at an operating voltage of 10 kV. Raman spectroscopy was conducted using a multichannel Raman imaging system (ST Japan Inc., Japan) at an incident wavelength of 532 nm. Transmission electron microscopy (TEM) and scanning TEM (STEM) images were captured using a JEM-ARM200F NEOARM TEM/STEM (JEOL Ltd., Japan) equipped with an EDX spectrometer operating at 200 kV. Double-spherical aberration (*C*_s_) correctors (CEOS GmbH, Heidelberg, Germany) were used to obtain high-contrast images with a lattice resolution of 0.7 Å. EDX profiles were collected using a JEOL JED-2300 T instrument. X-ray photoelectron spectroscopy (XPS) measurements were conducted using a JPS 9010 TR spectrometer (JEOL Ltd., Japan) equipped with a Mg Kα X-ray source (*λ* = 1253.6 eV). To that end, the pass energy was set to 10 eV, and each sample was placed on a piece of graphite tape. Because charge accumulation in the sample shifted the binding energy to higher values, the charge-up magnitude was calibrated using the C 1s peaks of graphene and graphite tape at 284.6 eV.

### 2.6. Calculation of crystal size

The crystal size of the powder materials was calculated using the Scherrer Equation (2.1).

(2.1) $$\mathrm{Crystal}\;\mathrm{Size}=\frac{\mathrm{k\lambda}}{\beta \cos\theta },$$

where *k* is a dimensionless shape factor, typically considered as ~ 0.9; *λ* is the X-ray wavelength (1.54060 Å (Cu Kα_1_) in this study); *θ* is the incidence angle of X-ray with respect to the sample holder; *β* is the full width at half maximum intensity of the peak.

### 2.7. Electrochemical measurements

All electrochemical measurements were performed using a Corrtest CS2350H electrochemical workstation in aqueous 0.5 M H_2_SO_4_. The standard three-electrode setup comprised an active-material-loaded glassy carbon electrode (GCE) as the working electrode, a carbon rod as the counter electrode, and Ag/AgCl as the reference electrode. Except for the active material, all the electrodes were purchased from BAS Inc., Japan. For each measurement, the prepared ink (15 μL) was dropped thrice onto the polished surface of the GCE to achieve a final catalyst loading of 0.05 mg cm^−2^. Linear sweep voltammetry (LSV) curves were recorded at a scan rate of 5 mV s^−1^.

All experiments were conducted at ambient temperature (298 ± 2 K), and the potentials were evaluated against the reversible hydrogen electrode (RHE) using Equation (2.2).

$$E_{\mathrm{RHE}}=E_{\mathrm{Ag}/A\mathrm{gCl}}+0.059\times \mathrm{pH}+E_{Ag/\mathrm{AgCl}}^{0}$$

$$=E_{\mathrm{Ag}/A\mathrm{gCl}}+0.059\times 0+E_{Ag/\mathrm{AgCl}}^{0}$$

(2.2) $$=E_{\mathrm{Ag}/A\mathrm{gCl}}+0.199V\left(\mathrm{at}\;\mathrm{pH}0\right),$$

where *E*_Ag/AgCl_ is the measured potential, and *E*^0^_Ag/AgCl_ is the standard potential of Ag/AgCl (saturated KCl) at 25°C (0.199 V).

The thermodynamic potential for the HER is 0 V; therefore, the absolute value of the potential obtained by LSV is the overpotential. To better elucidate the HER kinetics, Tafel slopes were derived from the LSV plots. To evaluate the electrocatalytic stability of the catalysts, the LSV curves before and after 6000 CV cycles (0.1 to −0.3 V vs. RHE) at a scan rate of 100 mV s^−1^ were compared.

## 3. Results and discussion

### 3.1. Characterization of ball-milled MoS_2_

**Figure 1.**
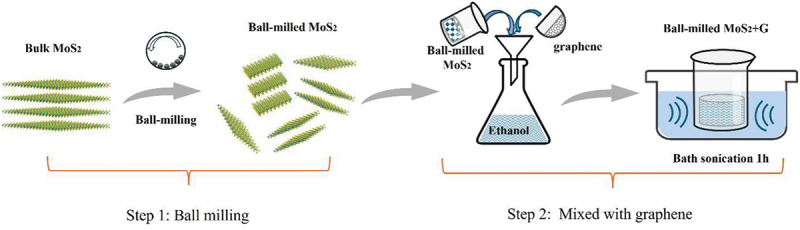
Synthesis of graphene-integrated ball-milled MoS_2_ electrocatalyst.

**Figure 2.**
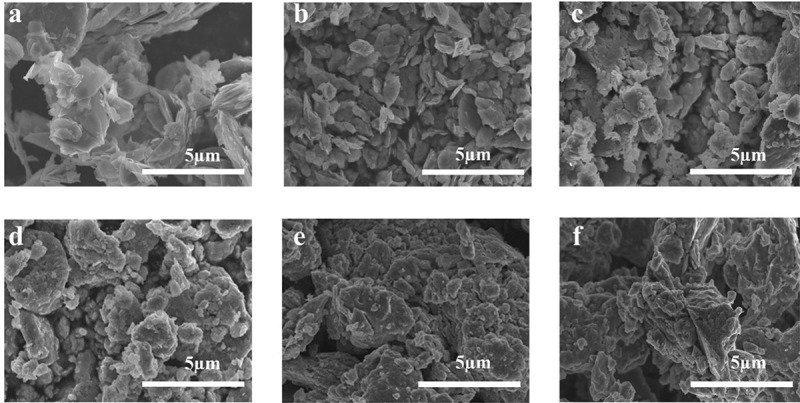
SEM images of (a) pristine bulk MoS_2_, (b) MoS_2_-30 min, (c) MoS_2_-2 h, (d) MoS_2_-4 h, (e) MoS_2_-8 h, and (f) MoS_2_-12 h.

**Figure 3.**
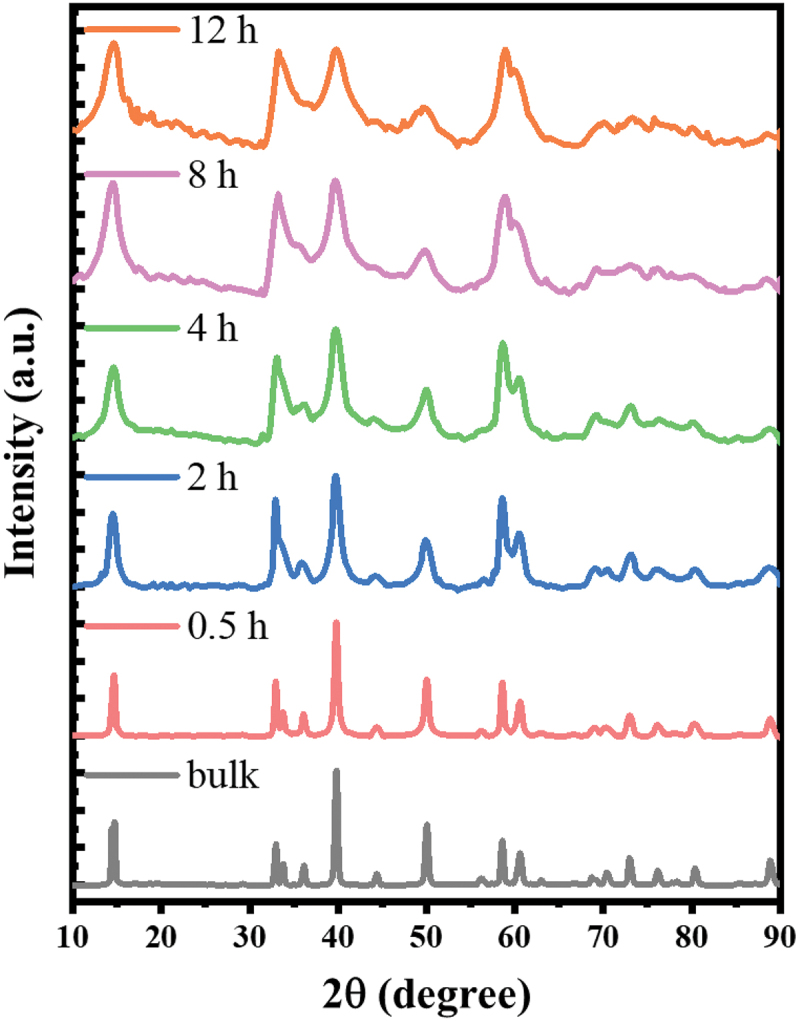
XRD patterns of bulk MoS_2_ powder and MoS_2_ samples ball-milled for different durations.

Ball milling is a straightforward, effective method for exfoliating and decreasing the lateral dimensions of large quantities of layered materials. As shown in Figure 1, an industrial milling machine was used to produce small flakes of MoS_2_ with different thicknesses, starting from large grains of natural MoS_2_, followed by the addition of graphene to increase conductivity and realize superior electrocatalytic performance. SEM was performed to examine the milling-induced morphological changes. As shown in Figure 2, smaller MoS_2_ particles were produced via ball milling. Furthermore, XRD was conducted to evaluate the crystal structure and crystal size of each sample (Figure 3), with the Scherrer equation used for the calculations. The results (Table 1) indicated that there was no appearance of the other crystal structure, and the crystal size of MoS_2_ gradually reduced from 68 to 4 nm by ball milling.

**Table 1.** Crystal sizes of MoS_2_ samples ball-milled for varying periods, calculated using the Scherrer equation.

Sample	2*θ* (degree)	FWHM	Crystallite size (nm)
Bulk MoS_2_	13.63	0.12	68
MoS_2_-15 min	14.05	0.23	35
MoS_2_-30 min	14.03	0.30	26
MoS_2_-1 h	14.06	0.51	15
MoS_2_-2 h	14.05	0.90	8
MoS_2_-4 h	14.06	0.98	6
MoS_2_-12 h	14.05	1.20	4

**Figure 4.**
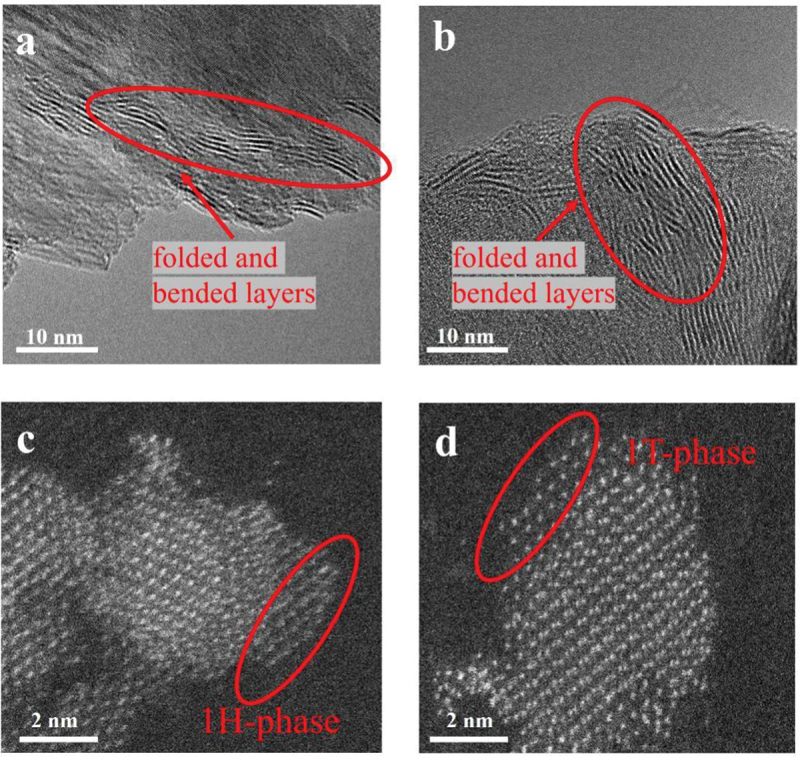
STEM images of (a) MoS_2_-30 min and (b – d) MoS_2_-12 h.

High-resolution STEM was performed to further evaluate the structural properties of ball-milled MoS_2_. As shown in Figure 4(a,b), the MoS_2_-30 min and MoS_2_-2 h samples were well crystallized and contained folded, bent layers. Notably, the 2 H phase is the most common and stable configuration of MoS_2_ [26]. When MoS_2_ is multi-layered, the most stable phase is the 2 H phase, and when it is single-layered, it is the 1 H phase [29]. The difference in the structures of the 1T and 1 H phases was identified by the contrast difference as reported previously [29]. The part captured in the photographs is single-layer MoS_2_; therefore, it is the 1 H phase. According to the atomic structure model, the 1 H and 1T phases existed simultaneously in the MoS_2_-12 h sample (Figure 4(c,d)), suggesting that ball milling caused the formation of the 1T phase, presumably through exfoliation.

Further insights into the surface compositions of the materials were obtained by XPS. Specifically, the wide-scan compositional spectra of MoS_2_ (Figure S1) and the high-resolution Mo 3d and S 2p spectra (Figure S2) were acquired before and after milling for comparison. Notably, Si was detected in addition to Mo and S in the wide-scan spectra (Figure S1); Si originated from SiO_2_, which inevitably emerged during the XPS measurements as the samples were prepared by dripping the sample ink onto a glass slide. The high-resolution Mo 3d and S 2p XPS profiles (Figure S2) showed typical Mo 3d spectra for bulk MoS_2_, with sharp signals at approximately 229.5 and 232.6 eV, corresponding to Mo^4+^ 3d_5/2_ and Mo^4+^ 3d_3/2_, respectively. After ball milling, the MoS_2_-12 h sample exhibited a wider spectrum with peaks that shifted to higher energies. This can be ascribed to the presence of Mo atoms with higher oxidation states – that is, Mo^5+^ and Mo^6+^ [30,31]—which result from an oxidative process occurring during ball milling. Notably, a shift in the Mo 3d peaks toward higher energies has been recorded in the presence of high surface-step densities [32]. Ball milling likely produced several surface step edges and defects while reducing the lateral size of the thin film. Similar conclusions were drawn from the S 2p spectra acquired before and after ball milling: the appearance of oxidized higher peak components in S 2p results in broader peaks possibly owing to the oxidation of surface sulfur because of ball milling.

Elemental quantification was performed by acquiring narrow-scan XPS profiles, and the atomic percentage of each element was estimated (Table 2). The S/Mo ratio decreased slightly with increasing ball-milling time, and the longer the ball-milling time, the higher the sulfur defect; this implies that the milling did not induce a significant chemical transformation but created certain sulfur defects that potentially favored electrochemical reactions [33–35].

**Table 2.** Atomic percentages of the main elements detected by XPS for pristine bulk MoS_2_ and MoS_2_ samples ball-milled for different durations. The values of Mo and S are derived from the areas of the Mo 3d peak and S 2p peaks with sensitivities (Mo 3d5/2:23.52 and S 2p3/2:7.18) determined using SpecSurf software (JEOL Ltd., Japan).

Sample (MoS_2_)	Mo (at%)	S (at%)	S/Mo ratio	MoS_2_ (S/Mo: 2/1)
Bulk	44.6	55.4	1.24	2.00
Ball-milled 30 min	45.6	54.4	1.19	1.92
Ball-milled 1 h	46.5	53.5	1.15	1.85
Ball-milled 2 h	46.9	53.1	1.13	1.82
Ball-milled 4 h	47.5	52.5	1.10	1.77
Ball-milled 12 h	48.2	51.8	1.07	1.72

### 3.2. Electrochemical performance

The electrocatalytic performances of the prepared HER catalysts were tested in a 0.5 M N_2_-saturated H_2_SO_4_ solution at a rotating speed of 1600 rpm. The electrochemical performance analysis of MoS_2_ samples ball-milled for different durations, with respect to commercial Pt/C as the reference (Figure S3), showed that the polarization curve gradually shifted to the right after ball milling. This implied that the overpotential decreased, signifying that ball milling undeniably improved the catalytic activity of MoS_2_. However, samples ball-milled for longer durations did not always exhibit superior activity. In particular, the 8 h and 12 h ball-milled samples performed worse than the other ball-milled samples. Based on the information obtained from SEM, the smaller MoS_2_ particles produced after prolonged ball-milling agglomerated, which likely caused the exposed edges to be covered, thereby reducing the number of active sites and resulting in poor performance.

To disperse the agglomerated particles and thereby obtain catalysts with superior performance, four of the samples were subjected to an additional two hours of bath sonication. As shown in Figure S4 and Table S1, the properties of each sample improved after sonication. Moreover, the longer the ball-milling duration – or alternatively, the smaller the particles – the greater the improvement after the additional sonication. Sonication caused the agglomerated particles to redisperse, exposing more edges and thereby improving the catalytic activity. Furthermore, the samples ball-milled for longer durations exposed more edges than those of the samples subjected to shorter ball-milling periods; additionally, the ball-milling process produced the 1T phase (Figure 4) and sulfur defects (Table 2). These results are consistent with previous discussions on the active sites of MoS_2_; thus, the presence of more exposed edges, the 1T phase, and sulfur vacancies is imperative to the HER catalyzing activity [14,15,18].

CV measurements were conducted in the presence of ferro/ferricyanide cyanide as an oxidation – reduction probe to evaluate the heterogeneous electron transfer between the material and the ferro/ferri ions in the solution. As shown in Figure S5, the CV profile of MoS_2_-12 h differed only slightly from that of bulk MoS_2_. Notably, the oxidation and reduction current intensities of the redox probe increased by ~ 25%, presumably owing to the ball-milling-induced increase and decrease in the electrochemical surface area and particle size of the material, respectively. Additionally, slightly faster electron transfer occurred after ball milling, resulting in less peak-to-peak separation between the oxidation and reduction profiles of the reducing probe. This was also potentially due to the increased availability of the edge components, which have been proven to be remarkably potent active sites [36].

**Figure 5.**
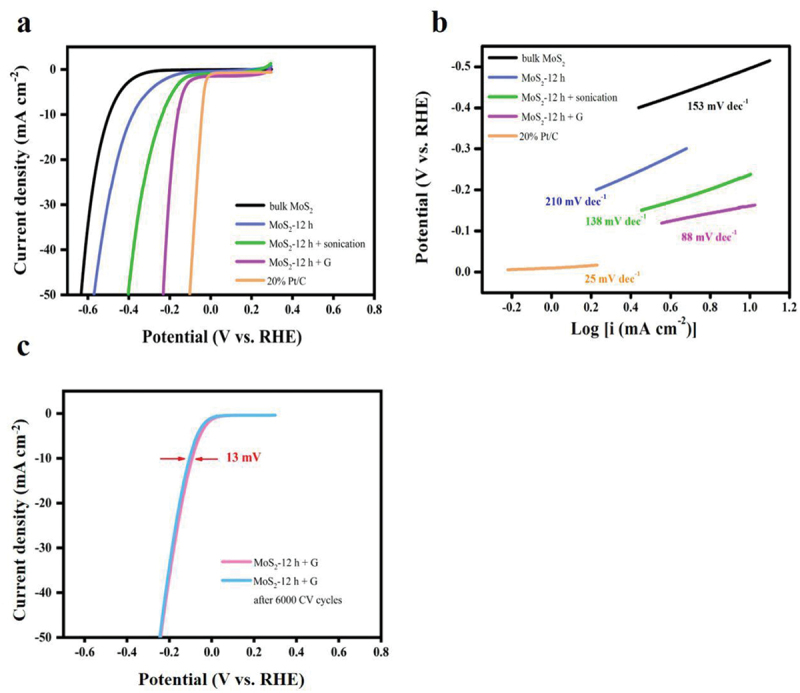
(a) LSV curves for HER with the as-prepared MoS_2_ samples in aqueous 0.5 M H_2_SO_4_, and (b) corresponding Tafel plots. (c) LSV curves of the MoS_2_-12 h + G electrocatalyst recorded before and after 6000 cyclic voltammetry cycles (0.1 to −0.3 V vs. RHE) in aqueous 0.5 M H_2_SO_4_.

The as-prepared MoS_2_ samples were subsequently integrated with graphene – a typical conductive material – to improve their activity further [37,38]; this also helped maintain the morphology of the exfoliated sheets. As shown in Figure S6 and Table S2, the mixture of ball-milled MoS_2_ and graphene (MoS_2_ + G) samples exhibited superior performance. In particular, the MoS_2_-12 h + G sample showed an overpotential (160 mV) that was ~335 mV lower than that of pristine bulk MoS_2_. This enhanced activity can be ascribed to the improved electronic conductivity and suppressed agglomeration of MoS_2_ particles by mixing with flexible graphene: as shown in Figure S7, an SEM-based morphological analysis of MoS_2_-12 h + G shows that graphene plays an important role in connecting MoS_2_ and the electrode as well as the spacer to prevent agglomeration. Furthermore, the only-graphene sample exhibited low activity, and comparing the CV cycles with a large range of potential (Figure S8), there is a distinct difference in the current densities between the only-graphene sample and MoS_2_-12 h + G sample, thereby confirming the important role of graphene in providing conductivity. A performance comparison was performed between bulk MoS_2_ and the sonicated and graphene-incorporated variants of MoS_2_-12 h (Figure 5 and Table S3). The MoS_2_-12 h + G sample exhibited improved performance, with a lower overpotential and a smaller Tafel slope. The decrease in the Tafel slope of MoS_2_-12 h + G may be due to the introduction of graphene-supported conductivity in the materials (in other words, the larger Tafel slope is due to low conductivity), and the smaller Tafel slope also indicates its superior kinetics. Based on this finding, the graphene addition had a better effect on the catalyst performance than sonication, presumably because graphene not only increased the electronic conductivity of the sample but also helped maintain the morphology and exposed edge state of MoS_2_ [39–41]. Furthermore, MoS_2_-12 h + G outperforms several other noteworthy MoS_2_-based electrocatalysts in the HER (Table S4 and Figure S9) [42–48]. Although certain catalysts exhibit better performance, they require additional and more complex procedures for operation. Therefore, the method used to prepare the graphene-integrated ball-milled MoS_2_ is more cost-effective than several other techniques [34,49–51].

Stability is another key factor in the evaluation of electrocatalysts. The electrochemical stability of MoS_2_-12 h + G was evaluated by comparing the LSV curves before and after 6000 CV cycles. As shown in Figure 5(c), the MoS_2_-12 h + G sample cycled 6000 times continued to exhibit high catalytic activity, and the overpotential at 10 mA cm^−2^ shifted by only 15 mV, suggesting its superior stability. Furthermore, the morphology of the MoS_2_-12 h + G sample did not change after the stability measurements (Figure S10). The surface elemental composition after 6000 CV cycles was evaluated by XPS (Figure S11), and the results revealed only a slight shift in the peaks after the long-term stability measurements, which were evidently due to slight oxidation on the surface. Collectively, these findings underscore the remarkable potential of the electrochemically stable graphene-incorporated ball-milled MoS_2_ for future applications.

## 4. Conclusion

In this study, the size of MoS_2_ particles was controlled using a simple mechanical process – ball milling – and the as-prepared samples exhibited significantly enhanced electrochemical and catalytic properties compared to those of pristine bulk MoS_2_. Upon ball milling, a 25% enhancement was observed in the current intensity, and peak-to-peak separation was achieved. The HER activity of the as-prepared ball-milled sample was further improved by introducing graphene. In particular, the MoS_2_-12 h + G sample exhibited superior performance, showing an overpotential (160 mV) that was ~335 mV lower than that of pristine bulk MoS_2_. These improved performance metrics were most likely due to the increased availability and density of edge planes in the material, which was achieved by the particle size reduction, the appearance of the 1T phase, and an increase in sulfur vacancies, in addition to the superior bolstering effect of graphene. Therefore, ball milling with graphene mixture shows promise as a new, scalable method for preparing size-controlled, highly active electrocatalysts.

## Supplementary Material

Supplemental Material
