# Supplementary material for: Ball-milled MoS2 with graphene shows enhanced catalytic activity for hydrogen evolution reaction
Source: Sci Technol Adv Mater. 2024 May 29;25(1):2359360. doi: 10.1080/14686996.2024.2359360 (PMC11177716; doi:10.1080/14686996.2024.2359360)
Supplement: Supplemental Material [file TSTA_A_2359360_SM6593.pdf]

Supplementary information

Ball-milled MoS_2 with graphene shows enhanced catalytic activity for hydrogen evolution reaction

Table of contents

Figures S1–S10 ………………………………………………… 1

Tables S1–S4 …………………………………………………… 8

Supplementary references ………………………..……..… 10

Supplementary figures

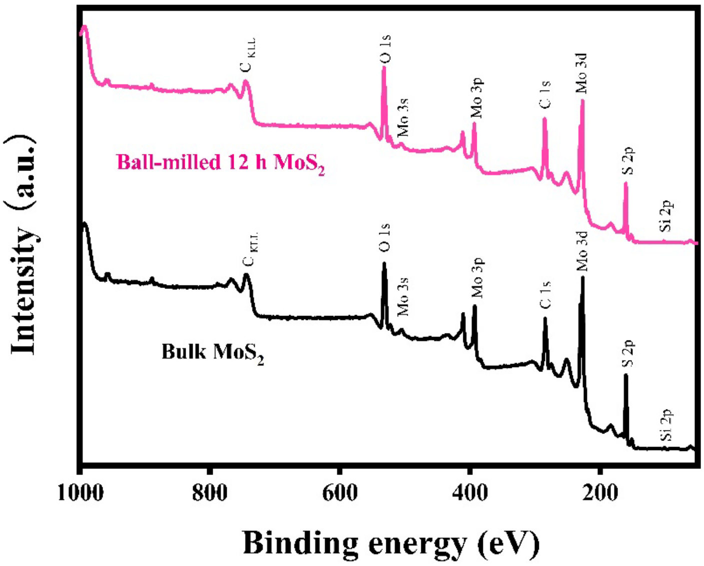

Figure S1. Wide-scan XPS profiles of bulk MoS_2 before (black) and after (pink) ball milling for 12 h. (Si originates from SiO_2, which is inevitably present during the XPS measurements, as each sample is prepared by dripping the sample ink onto a glass slide.)

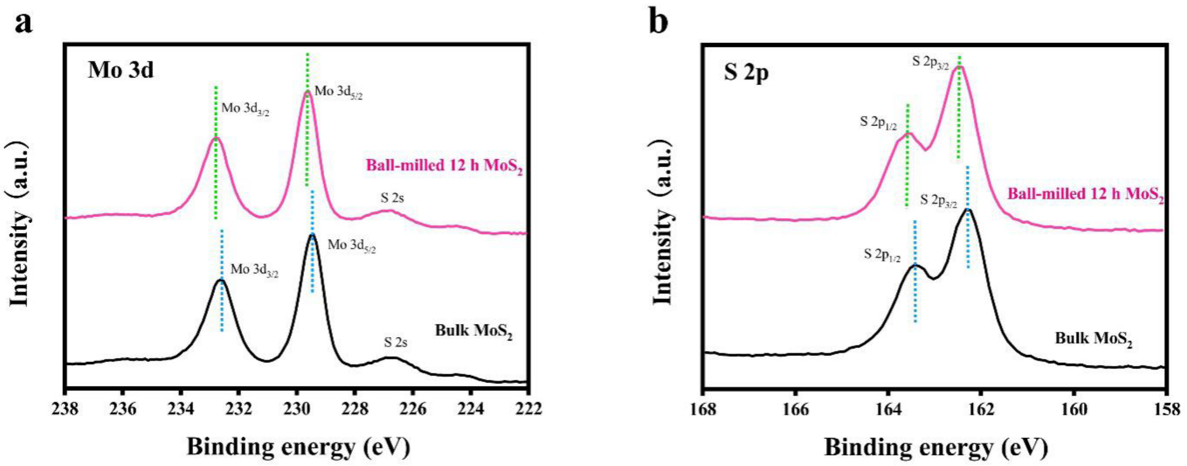

Figure S2. High-resolution (a) Mo 3d and (b) S 2p XPS profiles of bulk MoS_2 before (black) and after (pink) 12 h of ball milling.

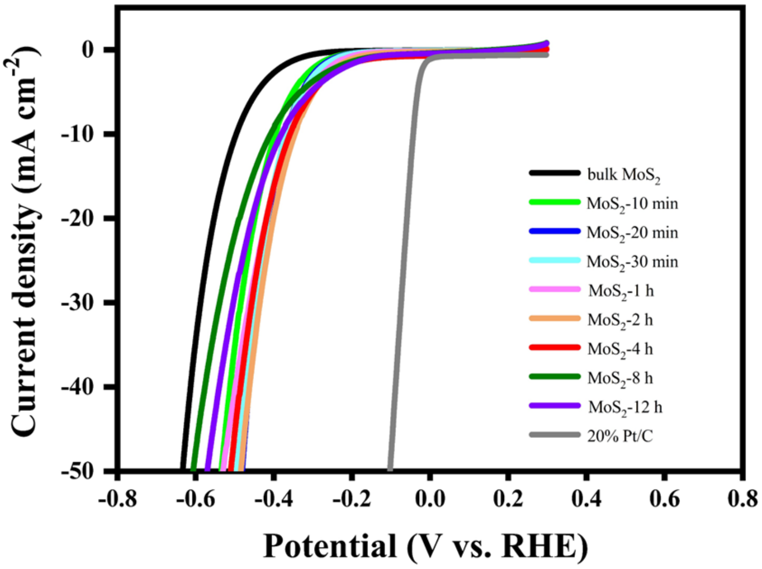

Figure S3. LSV curves of as-prepared ball-milled samples, a commercial 20% Pt/C specimen, and bulk MoS_2 in aqueous 0.5 M H_2SO_4.

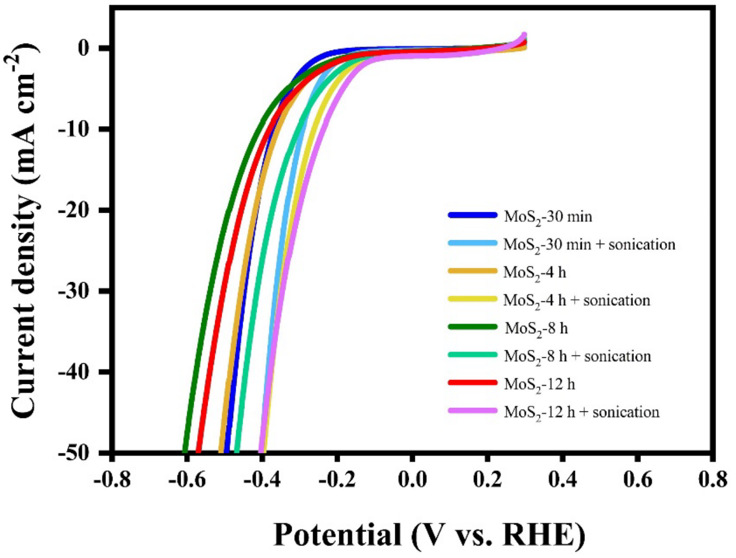

Figure S4. LSV curves for the HER of as-prepared and sonicated samples in aqueous 0.5 M H_2SO_4.

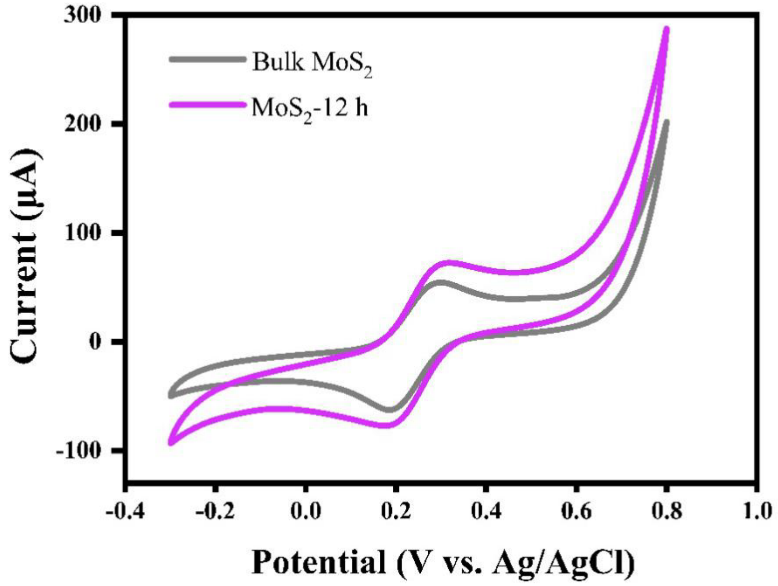

Figure S5. Cyclic voltammograms of 5 mM $[Fe(CN)_6]^{4-/3-}$ (vs. Ag/AgCl reference electrode) using bulk MoS_2 and MoS_2 ball-milled for 12 h, with 0.1 M KCl as the supporting electrolyte.

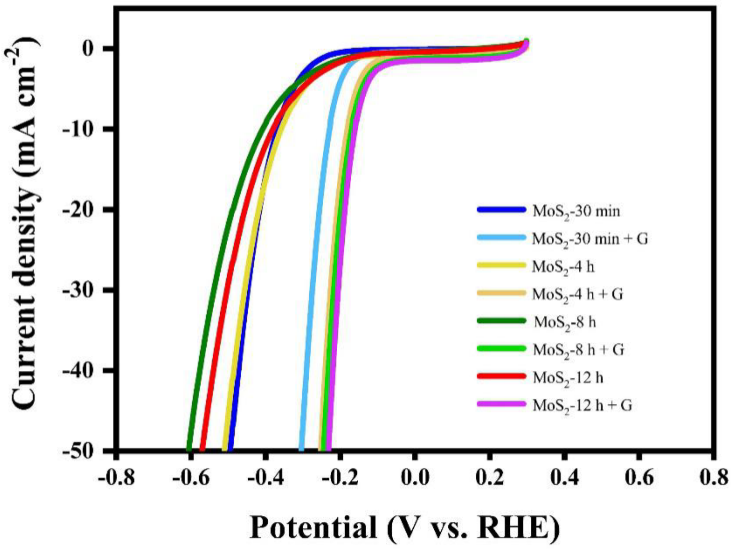

Figure S6. LSV curves for the HER of as-prepared and graphene-incorporated samples in aqueous 0.5 M H_2SO_4.

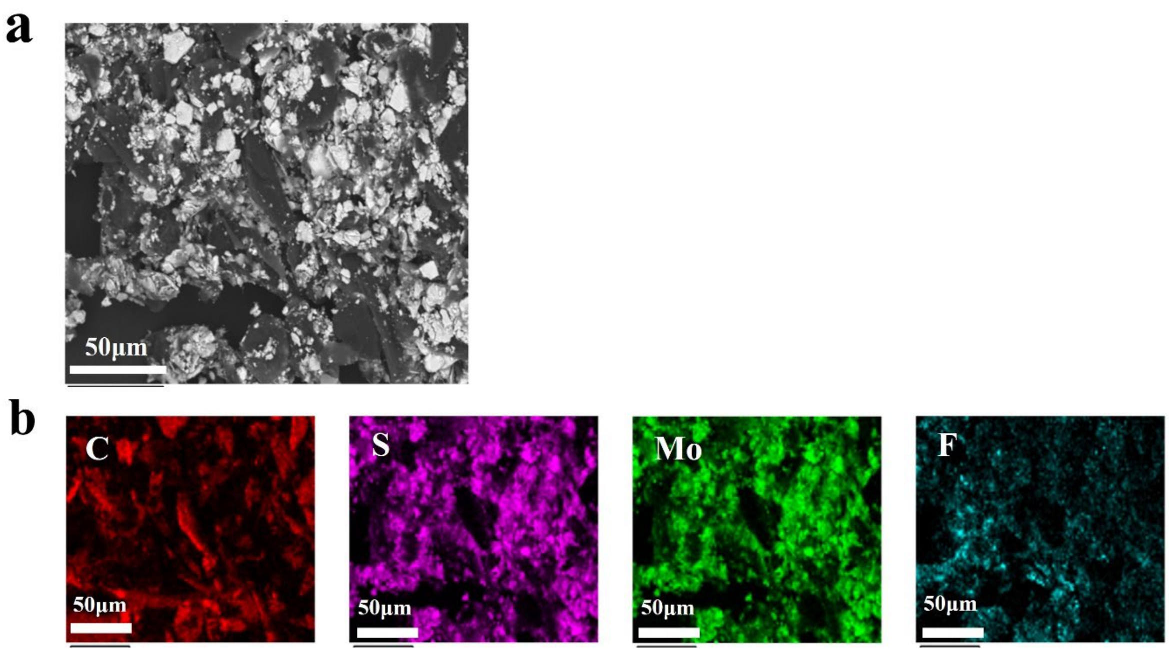

Figure S7. (a) SEM image and (b) electron-probe microanalysis mapping images of MoS_2-12 h + G with Nafion.

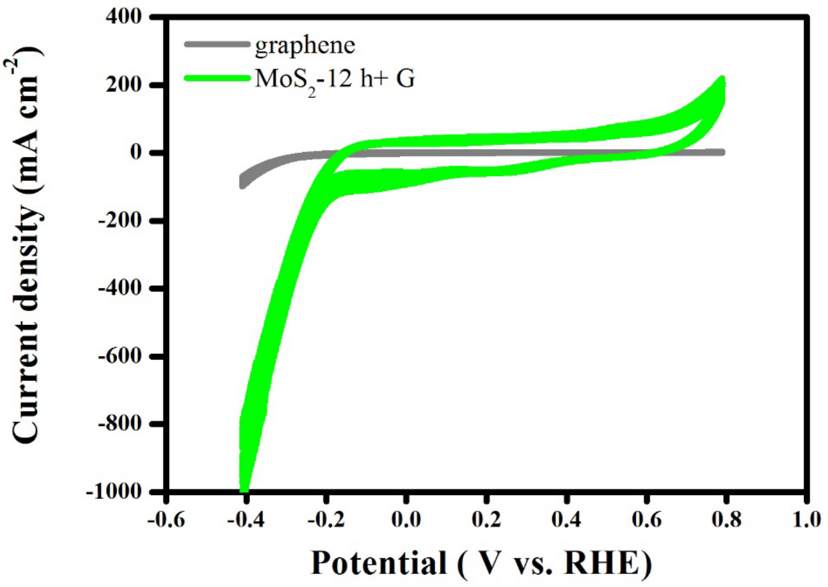

Figure S8. Cyclic voltammograms of graphene and MoS_2 -12 h + G sample in aqueous 0.5 M H_2SO_4.

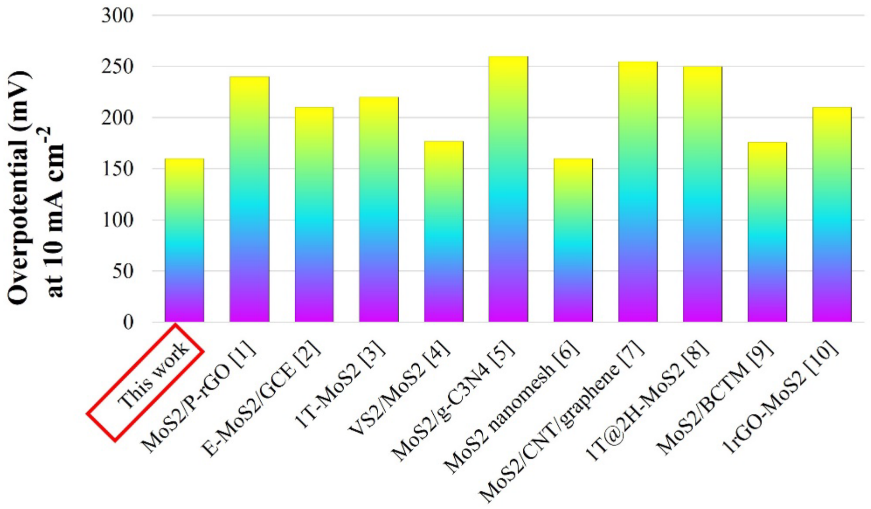

Figure S9. Overpotentials of several MoS_2-based catalysts at a current density of 10 mA cm^{-2} under acidic conditions [1–10].

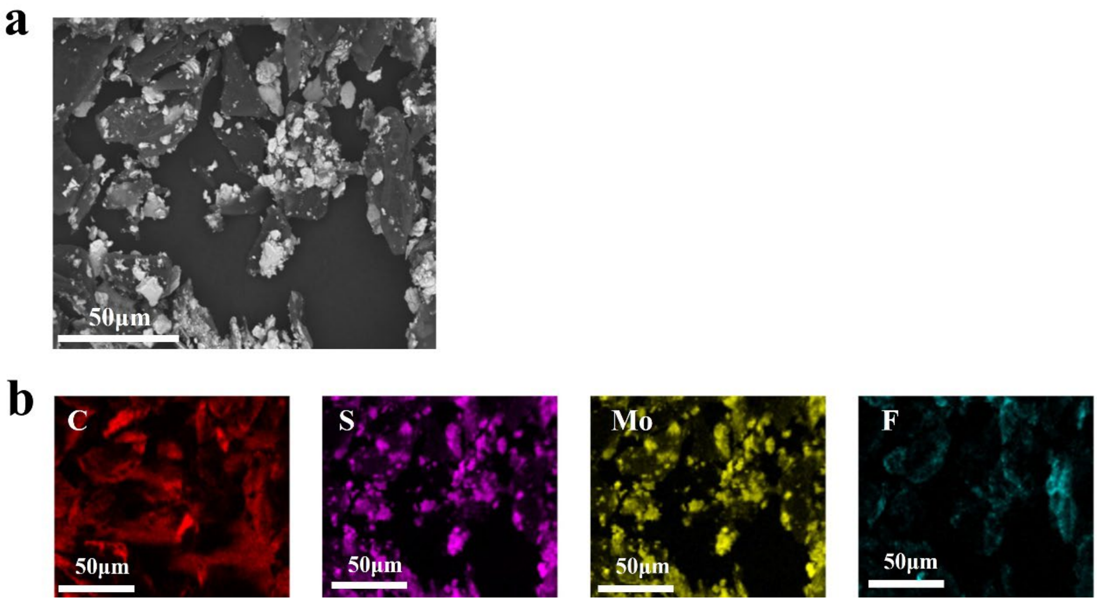

Figure S10. (a) SEM image and (b) electron-probe microanalysis mapping images of MoS_2-12 h + G with Nafion after stability measurements.

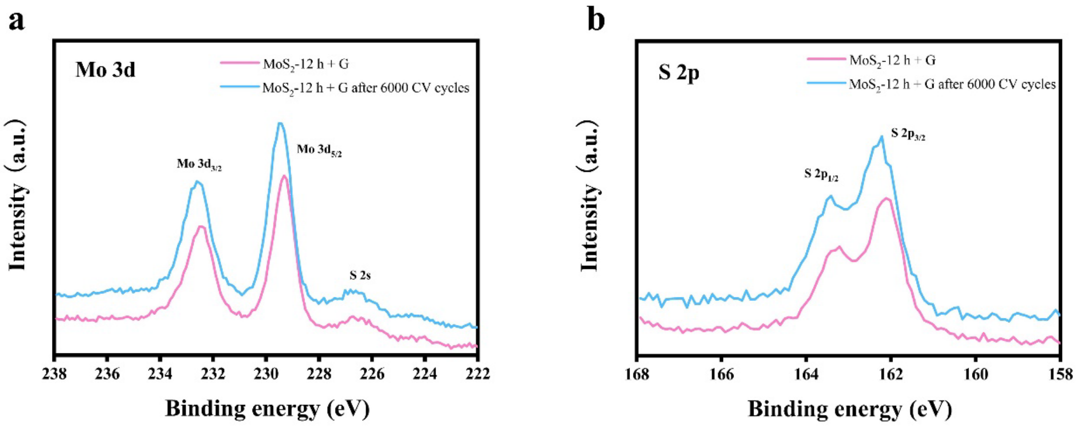

Figure S11. High-resolution (a) Mo 3d and (b) S 2p XPS profiles of MoS_2 ball-milled for 12 h before (pink) and after (blue) 6000 CV cycles.

Supplementary tables

Table S1. Comparison of as-prepared and sonicated samples

Sample	Overpotential at 10 mA cm^{-2} (mV)		Sample (with 2 h sonication)	Overpotential at 10 mA cm^{-2} (mV)
**MoS_2-30 min**	368		**MoS_2-30 min + sonication**	292 (76▼)
**MoS_2-4 h**	359		**MoS_2-4 h + sonication**	262 (97▼)
**MoS_2-8 h**	408		**MoS_2-8 h + sonication**	304 (104▼)
**MoS_2-12 h**	380		**MoS_2-12 h + sonication**	237 (143▼)

Table S2. Comparison of as-prepared and graphene-integrated samples.

Sample	Overpotential at 10 mA cm^{-2} (mV)		Sample (mixed with graphene)	Overpotential at 10 mA cm^{-2} (mV)
**MoS_2-30 min**	368		**MoS_2-30 min + G**	228 (140▼)
**MoS_2-4 h**	359		**MoS_2-4 h + G**	180 (179▼)
**MoS_2-8 h**	408		**MoS_2-8 h + G**	169 (239▼)
**MoS_2-12 h**	380		**MoS_2-12 h + G**	160 (220▼)

Table S3. Comparison of certain as-prepared, and sonicated, and graphene-incorporated samples

Sample	Overpotential at 10 mA cm^{-2} (mV)	Tafel slope (mV dec^{-1})
**Bulk MoS_2**	495	146
**MoS_2-12 h**	380	200
**MoS_2-12 h + sonication**	237	129
**MoS_2-12 h + G**	160	86
**20% Pt/C**	44	25

Table S4. Comparison of the HER performances of previously reported MoS_2-based electrocatalysts in acidic solutions.

	Overpotential at 10 mA cm^{-2} (mV)	Tafel slope (mV dec^{-1})	Reference
**MoS_2-12 h + G**	160	86	This work
**MoS_2/P-rGO**	240	75	[1]
**E-MoS_2/GCE**	210	70	[2]
**GQDs-MoS_2**	200	43	[3]
**1T-MoS_2**	220	61	[4]
**VS_2/MoS_2**	177	55	[5]
**MoS_2/g-C_3N_4**	260	63	[6]
**MoS_2 nanomesh**	160	46	[7]
**MoS_2/CNT/graphene**	255	100	[8]

**1T@2H-MoS_2**	250	88	[9]
**MoS_2/BCTM**	176	51	[10]
**1rGO-MoS_2**	210	41	[11]
**MoS_2 nano islands**	248	84	[12]

References

[1] Liu Y, Liu J, Li Z, et al. Exfoliated MoS_2 with porous graphene nanosheets for enhanced electrochemical hydrogen evolution. Int J Hydrogen Energy. 2018;43(30):13946–13952.

[2] Ji S, Yang Z, Zhang C, et al. Exfoliated MoS_2 nanosheets as efficient catalysts for electrochemical hydrogen evolution. Electrochim Acta. 2013;109:269–275.

[3] Guo J, Zhu H, Sun Y, et al. Doping MoS_2 with graphene quantum dots: structural and electrical engineering towards enhanced electrochemical hydrogen evolution. Electrochim Acta. 2016;211:603–610.

[4] Liu Z, Gao Z, Liu Y, et al. Heterogeneous nanostructure based on 1T-phase MoS_2 for enhanced electrocatalytic hydrogen evolution. ACS Appl Mater Interfaces. 2017;9(30):25291–25297.

[5] Chen X, Yu K, Shen Y, et al. Synergistic effect of MoS_2 nanosheets and VS_2 for the hydrogen evolution reaction with enhanced humidity-sensing performance. ACS Appl Mater Interfaces. 2017;9(48):42139–42148.

[6] Fageria P, Sudharshan KY, Nazir R, et al. Decoration of MoS_2 on g-C_3N_4 surface for efficient hydrogen evolution reaction. Electrochim Acta. 2017;258:1273–1283.

[7] Li Y, Yin K, Wang L, et al. Engineering MoS_2 nanomesh with holes and lattice defects for

highly active hydrogen evolution reaction. Appl Catal B. 2018;239:537–544.

[8] Murthy AP, Madhavan J, Murugan K. Recent advances in hydrogen evolution reaction catalysts on carbon/carbon-based supports in acid media. J Power Sources. 2018;398:9–26.

[9] Yao Y, Ao K, Lv P, et al. MoS_2 coexisting in 1T and 2H phases synthesized by common hydrothermal method for hydrogen evolution reaction. Nanomaterials. 2019;9(6):844.

[10] Qiao S, Zhao J, Zhang B, et al. Micrometer–scale biomass carbon tube matrix auxiliary MoS_2 heterojunction for electrocatalytic hydrogen evolution. Int J Hydrogen Energy. 2019;44(60):32019–32029.

[11] Joyner J, Oliveira EF, Yamaguchi H, et al. Graphene supported MoS_2 structures with high defect density for an efficient HER electrocatalysts. ACS Appl Mater Interfaces. 2020;12(11)12629–12638.

[12] Chen B, Hu P, Yang F, et al. In situ porousized MoS_2 nano islands enhance HER/OER bifunctional electrocatalysis. Small. 2023;19(14):2207177.
